# Clean-Label Starch Modifications: Dry Heat Treatment in Combination with Ion Exchange

**DOI:** 10.3390/foods15020246

**Published:** 2026-01-09

**Authors:** Johanna A. Thomann, Michael Polhuis, Jan O. P. Broekman, Hero J. Heeres, André Heeres

**Affiliations:** 1 Research Centre Biobased Economy, Hanze University of Applied Sciences, Zernikeplein 11, 9747 AS Groningen, The Netherlands; 2 Green Chemical Reaction Engineering, Engineering and Technology Institute Groningen, University of Groningen, Nijenborgh 3, 9747 AG Groningen, The Netherlands; 3 Innovation Hub East Groningen, Billitonweg 1, 9640 AE Veendam, The Netherlands; 4 Royal Avebe Innovation Center, Zernikelaan 8, 9747 AA Groningen, The Netherlands

**Keywords:** dry heat treatment, potato starch, clean label, mineral enrichment

## Abstract

Potato starch offers the unique potential of mineral enrichment through the presence of phosphorylated amylopectin chains. This property was utilised in a straightforward dual modification of native potato starch by combining mineral enrichment with dry heat treatments (DHT). DHT itself (110–130 °C, 3–6% moisture, 2 h) affords potato starches with lower viscosity and gelatinisation temperatures and higher contents of digestible starch. Prior ion exchange with Na^+^, K^+^, Mg^2+^, and Ca^2+^ enhanced the versatility of dry heat treatments. This study demonstrates the fine-tuning of functional properties (rheology) of these novel, dual-modified starches. Of special interest are magnesium and calcium due to their nutritional value and their valency, allowing ionic cross-linking. The present study contributes to the understanding of starch–ion interactions in DHT, clearly highlighting the role of specific ion effects, as per the Hofmeister series (K^+^ > Na^+^ and Ca^2+^ > Mg^2+^), in addition to the reversible ionic cross-linking effect of divalent cations. This knowledge is of use for potential substitution of chemically modified starches in food products, serving relevant trends and needs of today’s food industry for clean-label starches.

## 1. Introduction

Starch is a highly versatile biopolymer that finds utility in various applications [1]. Traditionally, the food sector first comes to mind, and, indeed, modified starches provide the right texture for many food types, including baked goods, soups, sauces, and dairy products, as well as plant-based foods [2,3]. Chemical starch modifications enable tweaking of pasting and gelling behaviour to achieve the desired effect in food products [4,5,6,7,8]. However, the use of chemicals requires labelling of the modified starches, for example, with an E-number in the European Union. Consequently, recent years have seen a rapidly increasing demand for modified starches that do not require labelling, sometimes referred to as clean-label starches [9].

Common clean-label starch modifications that have received considerable attention in the literature and are moreover amenable to commercial-scale application are annealing, heat–moisture treatment, and dry heating [10,11,12,13]. Annealing involves the thermal treatment of granular starch in excess water at a temperature below the starch gelatinisation temperature. As its name implies, annealing reduces crystalline defects within the semi-crystalline starch granules via restructuring of starch amylopectin chains [14]. Heat moisture treatment involves heating of granular starch at a temperature above its gelatinisation temperature but with a limiting amount of water such that gelatinisation does not occur. Here also, the treatment causes internal molecular rearrangements of the amylopectin chains [12]. Dry heating, in turn, involves heating near-anhydrous starch at elevated temperatures, typically greater than 110 °C, and usually yields an overall reduction in viscosity, as well as a decrease in gelatinization temperatures, by disrupting the supramolecular structure of granular starches [13,15,16,17].

In the case of potato starch as a substrate, an additional parameter enabling further refinement of final product properties is the bonded phosphate monoesters located primarily on the amylopectin chains. By changing the nature and valency of the cations associated with the negatively charged phosphate groups, starch pasting and other properties can be manipulated. Indeed, the influence of ion exchange has been reported previously in the case of annealing and heat–moisture treatment [18,19]. In contrast, the influence of (altered) mineral composition during dry heat treatment has been underreported in the literature, with only one study mentioning the use of salt in combination with dry heat treatment [20]. This is despite the potential health benefits that may be realised from mineral enrichment and the ease of fine-tuning pasting properties using this approach. Mineral enrichment of potato starch with vital minerals like magnesium or calcium ions is interesting in the context of food fortification [21,22], as well as for expanding the range of product properties that can be obtained with the starch modification at hand. Studying combinations of ion exchange and dry heat treatment in greater detail is therefore of interest.

The present study focuses on the dual modification of native potato starch through dry heat treatment and ion exchange, aiming to provide more fundamental insight into starch–ion interactions, the resulting physicochemical properties, and their potential as novel clean-label starches for food industries.

Magnesium and calcium enrichment is of special interest due to their dual positive charges, which permit ionic cross-linking, with the added benefit of enhanced nutritional aspects of the final starch products. Monovalent sodium and potassium enrichment is included to contrast the ionic cross-linking effect of divalent cations. Starch characterisations were carried out with a Rapid Visco Analyser, swelling power and solubility measurements, differential scanning calorimetry, X-ray diffraction, inductively coupled plasma optical emission spectroscopy, amylose determination, and a starch digestibility assay.

## 2. Methods

### 2.1. Chemicals

Native and drum-dried potato starches were provided by Royal Avebe (Veendam, The Netherlands). Food-grade MgCl_2_·6 H_2_O and CaCl_2_ with a purity of 99% and 94–96%, respectively, were provided by Nedmag (Veendam, The Netherlands). KCl and NaCl (Acros Organics, Geel, Belgium) had a purity of 99+% and 99.5%, respectively. All chemicals and enzymatic assay kits were used as received unless otherwise indicated. For differential scanning calorimetry and inductively coupled plasma optical emission spectroscopy measurements, calibration standards were the same as previously reported [18,19].

### 2.2. Ion Exchange Procedure

Ion exchange of native potato starch was performed as in our previous work [18,19]. The concentrations of the salt solutions in demineralised water were 1% (*w*/*w*) for CaCl_2_, 2% (*w*/*w*) for MgCl_2_·6 H_2_O, and 5% (*w*/*w*) for both NaCl and KCl solutions. A starch suspension (25% (*w*/*w*) dry starch in salt solution) was stirred for 3 h at room temperature before filtering in vacuo, followed by washing with demineralised water (3 times the amount of starch dry weight). The recovered starch was dried in a ventilation oven overnight at 35–40 °C. The amount of starch used was corrected for moisture content, which was determined using a moisture content analyser (Mettler Toledo HE73, Mettler Toledo B.V., Tiel, The Netherlands).

This ion-exchanged potato starch was used for the second modification step, which is dry heat treatment. Additionally, experiments in reverse order were performed to test whether the order of the dual modification matters. Here, dry-heated native potato starch was subjected to ion exchange as the second step of the dual modification.

### 2.3. Dry Heat Treatment (DHT)

The DHT conditions chosen in this study were comparable to typical dry heat treatments reported in the literature [23]. For the dry heat treatment (DHT), a pre-drying step at 40 °C was used to reduce the moisture content of native potato starch and ion-exchanged native potato starch to ca. 6–7.5% (*w*/*w*). Subsequently, ca. 17 g of starch (wet weight) was transferred into crystallisation dishes and placed in an oven (Memmert VO 500, PMP 500, Memmert, Schwabach, Germany). The target temperature of 110 °C (DHT 110) was reached within 30 min, and then, the temperature was held at the target temperature for 2 h. The samples were removed from the oven, covered with pierced tin foil, and cooled to room temperature in a fume hood. An additional series of dry-heated native potato starch was prepared by further reducing the initial moisture content to ca. 3% (*w*/*w*) and increasing the temperature to 130 °C (DHT 130). As before, dry heat treatment was conducted for 2 h on native potato starch with and without prior Mg- and Ca- exchange. The dual-modified starches and controls were obtained as white, granular powders.

### 2.4. Starch Characterization

#### 2.4.1. Inductively Coupled Plasma Optical Emission Spectroscopy (ICP-OES)

The method for analysing mineral composition by ICP-OES was identical to our previous work [18,19]. For the determination of Na, K, Mg, and Ca, 5 g of starch (exact weight and moisture noted) were ashed at 500–550 °C for 1 h. The ash was then refluxed in 80 mL of ultrapure water with 16 mL of aqua regia for 30–45 min. For the phosphorus determination (1 g, exact weight and moisture noted), the ashing step was skipped. After cooling to room temperature, the samples were diluted to 100.00 mL and transferred to ICP test tubes using a syringe with filters to avoid contamination with ash particles. The samples and four calibration standards were then measured by ICP-OES (Agilent Technologies 5110, Autosampler SPS4, Agilent Technologies, Santa Clara, CA, USA). To establish the reproducibility of cation determination, native potato starch was measured five times. The reproducibility of phosphorus determination was established by measuring native potato starch in duplicate.

#### 2.4.2. Amylose Titration

Amylose contents were determined by iodine complex formation as reported by Duan et al. [24]. Ca. 400 mg of starch sample (exact weight and moisture noted) was dispersed in 40 mL of demineralised water. An amount of 10 mL of 5 M NaOH solution was added, and the mixture was stirred for ca. 15 min until complete dissolution of the starch. The solution was quantitatively transferred to a volumetric flask, adjusting the volume to 100 mL with demineralised water. After homogenization, 25 mL of the starch solution was transferred to a beaker, two drops of phenolphthalein indicator were added, and the solution was neutralised using 1 M HCl. Following neutralization, 1 mL of 1 M HCl and 28 mL of 0.5 M NaCl were added. Finally, 1 mL of 1 M KI was added before placing the beaker on the auto-titrator for titration with 0.83 mM of KIO_3_.

Titrations were performed in duplicate at room temperature using an 888 Titrando auto-titrator equipped with a combined platinum ring electrode, an 801 magnetic stirrer, and Tiamo 2.5 Workplace software (Metrohm AG, Herisau, Switzerland). The software recorded the titrant volume at the equivalence point and the amylose content (%). For statistical analysis, a one-way ANOVA and a Tukey HSD test were carried out with significance differences defined at *p* < 0.05.

#### 2.4.3. X-Ray Diffraction (XRD)

All samples were moistened to a value of 15.5% (*w*/*w*) using demineralised water prior to analysis. The long-range order was analysed using a Bruker Advance D8 Powder XRD (Bruker, Berlin, Germany) with theta–theta geometry. Measurements were performed between 3 and 30° 2θ (time/step = 0.65, increment = 0.03). Relative crystallinity was determined using Spectragryph optical spectroscopy software (Version 1.2.16.1) [25] by performing a baseline correction and comparing the integrals of the original amorphous and crystalline and baseline-corrected (crystalline) spectrum (25% coarseness, integration by area, 3–28° 2θ) as described in detail previously [18,19]. Relative crystallinity is defined as the ratio of crystalline peak area and area of the entire diffractogram, including the amorphous background.

#### 2.4.4. Differential Scanning Calorimetry (DSC)

Thermal properties were determined on a DSC25 (Thermo Fisher with Autosampler, Thermo Fisher Scientific, Eindhoven, The Netherlands) as reported earlier [18,19]. A suspension of the starch sample was prepared in a vial (20% starch on dry basis in demineralised water) and thoroughly mixed. A drop of the suspension (5 to 12 mg) was placed in a Tzero Pan with a syringe, sealed with Tzero Hermetic Lids (Thermo Fisher Scientific, Eindhoven, The Netherlands) using a crimper press, and measured directly. The sample pan was heated from 35 to 85 °C at a heating rate of 10 °C/min. The measurements were performed at least in duplicate. For statistical analysis, a one-way ANOVA and a Tukey HSD test were carried out with significance differences defined at *p* < 0.05.

#### 2.4.5. Swelling Power and Solubility (SWP and SOL)

The method, equipment, and calculation to determine swelling power and solubility of starch samples were reported earlier [18,19]. Swelling power and solubility were measured in triplicate at 70 °C and at 90 °C. For this, sample suspensions (2% starch on a dry basis in demineralised water) were heated for 30 min while shaking at 200 rpm, followed by rapid cooling in ice to room temperature. The suspension was then centrifuged at 3600 *g* for 40 min. The supernatant was separated carefully using Pasteur pipettes, and the gel-like residue was weighed. The ratio between the gel residue and the initial amount of sample was calculated (g/g of starch on dry weight basis) as the swelling power. The supernatant was pipetted off carefully into tared test tubes, and the test tubes were placed in a vacuum oven until all liquid was evaporated. The dry sediment was then weighed and used for the calculation of solubility. A one-way ANOVA and a Tukey HSD test were carried out for statistical analysis, with significance differences defined at *p* < 0.05.

#### 2.4.6. Rapid Visco Analyser (RVA)

The pasting curves and properties of starch samples (5% starch on dry basis in demineralised water) were recorded using a Rapid Visco Analyser (Perten Instruments, Stockholm, Sweden). Heating profile and standard measurement settings are shown below (Table 1). Reproducibility was established by measurement of native and waxy potato starch in triplicate.

#### 2.4.7. Starch Digestibility

Digestibility of the starch samples was determined using a resistant starch assay kit from Megazyme, following the procedure from Englyst et al., with some adaptations as reported earlier [18,19,26]. The calibration was performed using five concentrations of glucose standard in duplicate, and the GOPOD procedure was downscaled to use microtiter plates for incubation and measurement with a spectrophotometer. The calculations of rapid digestible starch (RDS) (measured after 20 min), slowly digestible starch (SDS) (measured after 2 h), total digestible starch (TDS), and resistant starch (RS) (measured after 4 h) were changed accordingly. Furthermore, additional aliquot samples were obtained at t(0) and t(end) (total digestible starch). The reproducibility was established by measuring native potato starch in duplicate.

## 3. Results and Discussion

### 3.1. Starch Modifications and Compositional Changes

The dual modification consists of an ion-exchange step followed by dry heat treatment (DHT) (Table 2). The mineral composition, amylose, and phosphorus content of the native potato starch used were in line with data in the literature (Table 3) [1,27]. Native potato starch was stirred in aqueous NaCl, KCl, MgCl_2_, or CaCl_2_ solution at room temperature (NPS Mg, NPS Ca, NPS Na, NPS K). This ion exchange increased the amount of the target mineral while depleting the levels of the other cations. As reported in previous work by our group, no leaching of amylose was observed during ion exchange [18]. The mineral composition of the ion-exchanged starting materials prepared for subsequent DHT is shown in Table 3.

Before DHT, NPS Mg, NPS Ca, NPS Na, and NPS K were dried to a moisture level of ca. 6% (*w*/*w*). At the same time, native potato starch (NPS) was dried to ca. 6% (*w*/*w*) alongside the ion-exchanged starch (exact moisture levels reported in Table 2). For the first dry heat treatment series, the starch was heat-treated at 110 °C for 2 h (DHT 110). The samples are noted as DHT 110 NPS, DHT 110 NPS Na, DHT 110 NPS K, DHT 110 NPS Mg, and DHT 110 NPS Ca.

To study the influence of the order of modifications, the dual modification was also carried out in reverse order, so that ion exchange was performed after the dry heat treatment. For this, ion exchange with MgCl_2_ or CaCl_2_ solution was performed using dry-heated native potato starch (DHT 110 NPS). These samples were labelled as ‘DHT 110 NPS Mg, reverse order’ and ‘DHT 110 NPS Ca, reverse order’, respectively. Here, mineral composition was determined after dual modification (Table 3). Dry heat treatment did not have an influence on the ion-binding capacity of potato starch.

**Figure A1:**
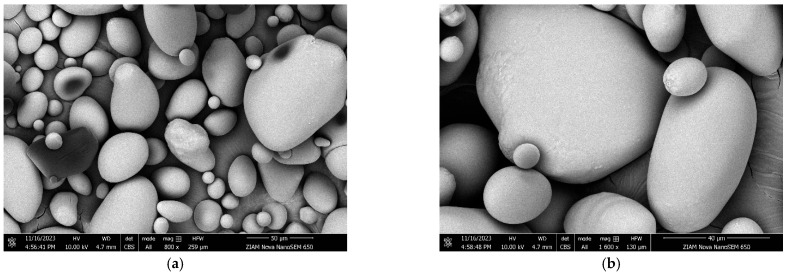
Scanning electron microscopy images of DHT 130 NPS (**a**) and (**b**).

To evaluate the effect of more severe conditions, a second dry heat series (DHT 130) was carried out using native potato starch and starch enriched with Mg- and Ca- ions. This means that NPS was used for DHT 110 NPS and DHT 130 NPS. NPS Mg was used for DHT 110 NPS Mg and DHT 130 NPS Mg. NPS Ca was used for DHT 110 NPS Ca and DHT 130 NPS Ca, as indicated in Table 2. For this series, the moisture content was further reduced to 2–3% (*w*/*w*), and the temperature was increased to 130 °C (Table 2). The samples are noted as DHT 130 NPS, DHT 130 NPS Mg, and DHT 130 NPS Ca. DHT 130 NPS was analysed for granular damage with scanning electron microscopy (SEM), and the granular surface remained intact (Figure A1).

Since DHT did not include a washing step, no changes in mineral composition are expected after DHT, since loss of Na^+^, K^+^, Mg^2+^, or Ca^2+^ cannot occur at 165 °C. To verify this, DHT 130 NPS, DHT 130 NPS Mg, and DHT 130 NPS Ca were measured after dry heat treatment (Table 3). Indeed, the levels of the target minerals remained constant with slight fluctuations. Changes in amylose content after DHT were controlled by analysing DHT 110 NPS, DHT 130 NPS, DHT 130 NPS Mg, and DHT 130 NPS Ca (Table 2). After DHT at 110 and 130 °C, amylose contents of 23.87 ± 1.11% and 23.84 ± 0.04% were found, respectively, which are not significantly different from the initial amylose content of the NPS of 23.97 ± 0.02%. Similarly, DHT 130 NPS Mg and DHT 130 NPS Ca yielded slightly higher but similar amylose readings of 24.36 ± 0.03% and 24.12 ± 0.06%, respectively.

The DHT conditions chosen in this study were comparable to typical dry heat treatments reported in the literature [23]. All dry-heated potato starches remained white and granular powders, while cation concentrations after ion exchange were in line with values reported in the literature [21,28,29].

### 3.2. Thermal Properties

**Figure 1:**
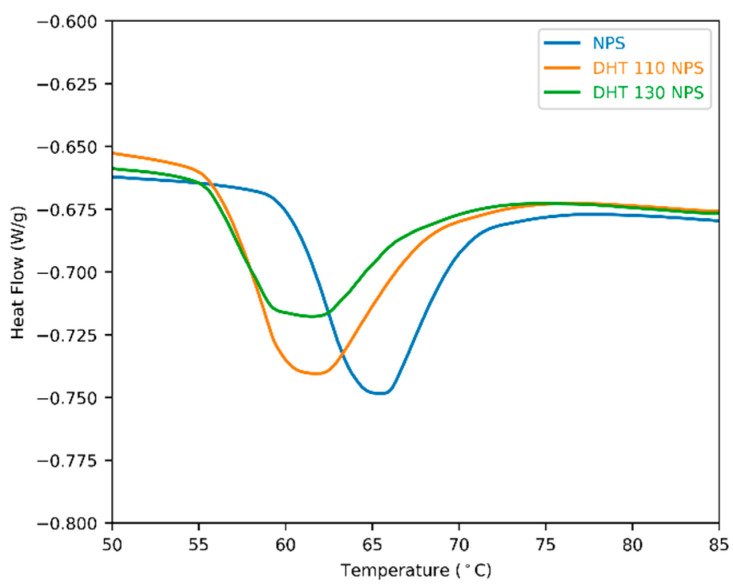
Thermogram of native potato starch (NPS) before and after dry heat treatment at 110 °C (DHT 110 NPS) and 130 °C (DHT 130 NPS).

Changes in crystalline structure can be analysed by differential scanning calorimetry (DSC). In general, dry heat treatment causes amorphisation of starch, which leads to lower gelatinisation temperatures and enthalpy [13]. Figure 1 below shows the thermograms of native potato starch and of native potato starch after DHT at 110 °C and 130 °C. Both treatments cause a similar shift towards lower temperatures, with a reduction of ca. 4 °C compared to NPS, together with peak broadening, indicating compromised crystalline structures. Harsher conditions during dry treatment reduced the onset temperature more strongly, as observed in DHT 130 NPS (Table 2).

In the DHT 110 series, dry heat treatment decreases the onset, peak, and conclusion temperatures compared to native potato starch (Table 4). An endothermic transition was observed in all cases, with the onset temperatures decreasing from highest to lowest in the following order:

DHT 110 NPS Ca > DHT 110 NPS Mg = DHT 110 NPS K > DHT 110 NPS Na > DHT 110 NPS

Combination with ion exchange is associated with slightly higher onset temperatures compared to the dry-heated native potato starch. DHT 110 NPS Ca showed the highest onset temperature, followed by DHT 110 NPS Mg and DHT 110 NPS K, for which the onset temperatures are not significantly different. Lastly, DHT 110 NPS Na showed the lowest onset temperature. Overall, however, the differences in onset temperatures do not exceed 1.7 °C, suggesting that the influence of ion exchange is relatively small. Therefore, it is difficult to draw conclusions on whether different cations accelerate amorphisation to different extents.

In contrast to the DHT 110 series, the results for the DHT 130 series reveal no significant difference in onset temperatures when Ca^2+^ is the dominant cation compared to Mg^2+^ ions. The presence of divalent cations during DHT 130 seems to increase the onset temperatures compared to the dry-heated native potato starch, as follows:

DHT 130 NPS Ca = DHT 130 NPS Mg > DHT 130 NPS

Notably, ionic cross-linking with divalent cations may not impact thermal properties strongly, since thermal transitions are mostly governed by processes occurring in the crystalline region. Phosphorylated glucose units are located in the amorphous region and influence granular swelling and amylose leaching. In DSC measurements, however, the gelatinisation peak arises mostly from the disruption of stacked double helices (crystalline melting) and helix-decoiling transitions.

In contrast, magnesium-enriched oxidised starches show higher gelatinisation temperature through ionic cross-linking due to binding of the abundant carboxyl groups and increased chain mobility through partial hydrolysis [30]. Here, the order within crystalline lamellae is already strongly disrupted due to the presence of shorter chains and introduced carboxyl groups. Moreover, if ions accelerate degradation processes like hydrolysis of glycosidic bonds or even complete or partial gelatinisation, DSC should be apt to reveal these ion effects.

In a study from 2021, Zhang et al. studied combinations of dry heat treatment of starch after prior calcium enrichment with 3% (*w*/*v*) CaCl_2_ solution (at 25 °C for 5 h) [20]. They observed higher gelatinisation temperatures after calcium enrichment of potato starch and connected these observations to ionic cross-linking effects; however, the observed differences were not significant. In their studies, calcium enrichment prior to dry heat treatment led to complete loss of crystallinity, where no thermal transition could be observed in the thermogram. However, moisture contents prior to dry heat treatment were not reported, making direct comparisons with the present results difficult. Another difference is that higher concentrations of CaCl_2_ were used. It could thus be that amorphisation of the starch granules may have occurred, reminiscent of surface gelatinisation in chemical peeling studies. However, this is speculative considering that peeling studies are generally performed using much higher concentrations of CaCl_2_ (>40%) [31].

### 3.3. X-Ray Diffraction

X-ray diffraction (XRD) allows insights into the long-range order of starch, which is known to be influenced by hydrothermal starch modifications [11,13]. The crystalline lamellae of starch granules are made up of stacked double helices of amylopectin chains, which can be arranged in different polymorphs. The hexagonal arrangement found in potato starch is called the B -type polymorph, with characteristic peaks at 5.5° 2θ, 17° 2θ, 22, and 24° 2θ [1,32].

**Figure 2:**
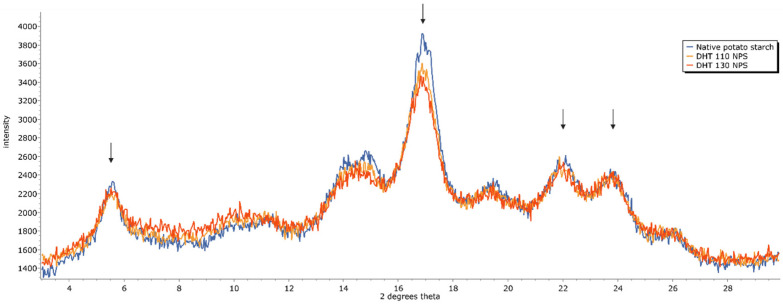
Diffractogram of native potato starch before and after dry heat treatment at 110 °C (DHT 110 NPS) and 130 °C (DHT 130 NPS). Characterisitic peaks for B-type crystallinity are highlighted.

**Figure 3:**
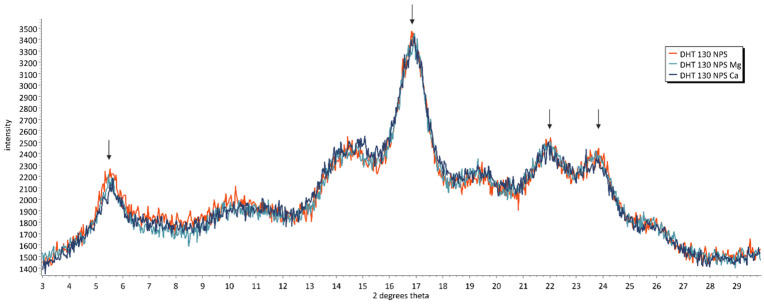
Diffractogram of dry-heated native potato starch (DHT 130 NPS) without and with prior ion exchange with magnesium (DHT 130 NPS Mg) or calcium ions (DHT 130 NPS Ca). Characterisitic peaks for B-type crystallinity are highlighted.

Dry heat treatment at 110 °C caused an overall broadening of the diffractogram, a decrease in peak height at 17° 2θ, and slight decreases in relative crystallinity (Figure 2). In DHT 130 NPS, further broadening and decreases in peak intensities were observed compared to those in DHT 110 NPS. Also, compared to NPS, the relative crystallinity values are lower by 1.8% points in DHT 110 NPS and by 2.3% points in DHT 130 NPS. No clear differences in DHT 130 NPS, DHT 130 NPS Mg, and DHT 130 NPS Ca could be observed (Figure 3), and relative crystallinity values were nearly the same, with differences between 0.3% and 0.6% points. The crystallinity type remained the same in all DHT samples.

The XRD data agree well with the thermal properties discussed in the previous section. Both the broadening observed in XRD spectra and the shift to lower gelatinisation temperatures provide evidence of heat-induced starch amorphisation, which was more severe with higher treatment temperature (DHT 110 vs. DHT 130). Moreover, XRD and DSC did not reveal obvious changes in starch crystallinity due to the presence of Mg^2+^ or Ca^2+^ ions during DHT.

The amorphisation of potato starch upon dry heat treatment supports the notion that DHT elicits controlled starch degradation, thereby generating minor structural changes as described by He et al. in a recent review on dry heat treatment [13]. Some groups reported a change from B-type to B + A-type crystallinity in waxy potato after dry heat treatment [23], which contrasts with our findings, where crystallinity type remains unaltered. However, in their study, no new peaks commensurate with A-type crystallinity were observed, and instead, only the characteristic peak at 5.5° 2θ was broadening and disappearing. Hence, the observed changes could also be a result of stronger overall degradation.

### 3.4. Swelling Power and Solubility

Further insights into starch swelling and its water-holding capacity, as well as solubility, can be obtained with swelling power measurements. Changes in starch crystallinity induced by hydrothermal modifications are expected to change these properties [8,11].

The swelling power of native potato starch remained similar after dry heat treatment at 110 °C or 130 °C, when measured at 90 °C (Table 5). Measured at 70 °C, swelling power was slightly increased in DHT 110 NPS and more so in DHT 130 NPS. However, large standard deviations were observed in DHT 110 NPS. Generally, when measured at higher temperatures, higher values for swelling power were obtained, as expected for this analysis [8,21]. DHT 110 NPS Mg and DHT 110 NPS Ca had similar but slightly lower SWP than DHT 110 NPS at both measurement temperatures. DHT 110 NPS Na and DHT 110 NPS K had a higher swelling power than DHT 110 NPS and NPS at both measurement temperatures. No differences in SWP between the monovalent and between the divalent cations were observed. DHT 130 NPS Mg and DHT 130 NPS showed a higher solubility than DHT130 NPS at both measurement temperatures, which could be a sign of slightly stronger amorphisation when divalent cations are present during harsh dry heat treatments. At 70 °C, DHT 130 NPS Mg and DHT 130 NPS Ca showed similar and slightly lower SWP compared to DHT 130 NPS. When measured at 90 °C, SWP of DHT 130 NPS Mg was lower than DHT 130 NPS and DHT 130 NPS Ca, in line with RVA measurements.

The tendencies of divalent cations suppressing swelling power and monovalent cations increasing this property are observable; however, these differences are not always significant. The influence of ions and ionic cross-linking is more evident in the initial swelling phase of gelatinisation. This is captured more clearly in RVA measurements, which will be discussed in the next section.

The obtained swelling power results in Table 4 are in line with earlier work by our group on combinations of ion exchange with annealing or heat–moisture treatment [18,19]. Moreover, Noda et al. reported a slight decrease in swelling power in native potato starch after ion exchange with calcium ions, matching our findings [28]. They attributed the changes in swelling power to ionic cross-linking effects.

Reports of the impact of dry heat treatment itself on swelling power are varied in the literature, depending on specific treatment conditions [13]. Zhang et al. reported increases in solubility and moderately lower swelling power of native potato starch after prolonged dry heat treatment due to chain degradation and amorphization [20]. In contrast, Liu et al. found increased swelling power values after dry heat treatment of waxy potato starch, as the more crystalline structure of waxy starch was easier to hydrate after partial amorphization [23].

The literature on the combined impact of ions and dry heat treatment is very limited. Zhang et al. reported an increased solubility and substantially decreased swelling power of dry-heated starches which were previously treated with concentrated CaCl_2_ solution for surface peeling [31].

### 3.5. Pasting Properties

**Figure 4:**
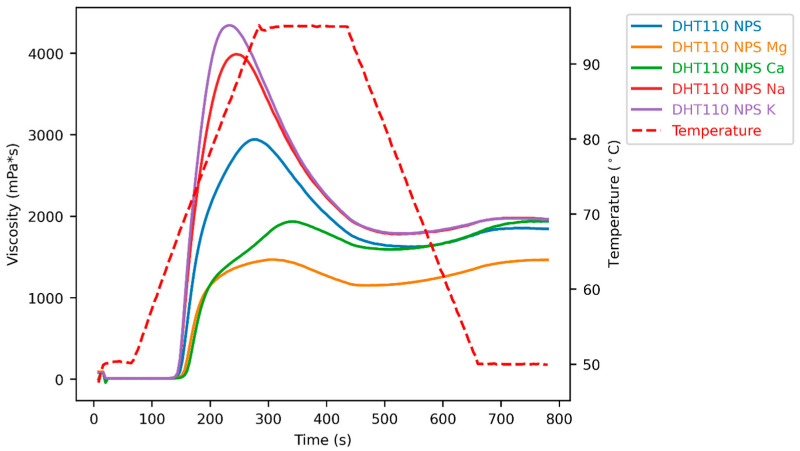
Pasting curves of native potato starch dry-heated at 110 °C (DHT 110 NPS) and dual-modified starches with prior ion exchange with sodium (DHT 110 NPS Na), potassium (DHT 110 NPS K), magnesium (DHT 110 NPS Mg), or calcium ions (DHT 110 NPS Ca).

**Figure 5:**
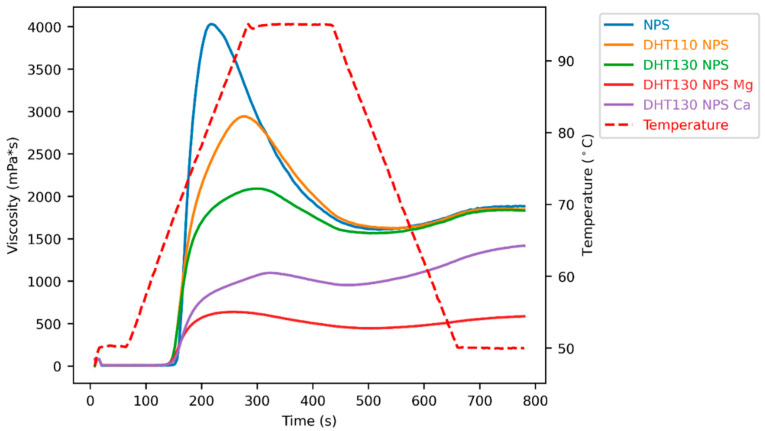
Pasting curves of both dry heat treatments (DHT 110 NPS and DHT 130 NPS), as well as combinations of dry heat treatment at 130 °C with ion exchange with divalent cations (DHT 130 NPS Mg and DHT 130 NPS Ca).

The pasting behaviour of starches is influenced by many factors, including granule crystallinity, amylose content, phosphate content, mineral composition, and measurement conditions [8]. Dry heat treatment itself decreased peak viscosity and pasting temperature at both treatment temperatures (DHT 110 NPS and DHT 130 NPS), while trough and final viscosity were almost unaltered compared to native potato starch (Figure 4 and Figure 5, Table A1). The dry-heated native granules are less shear stable, so that granular breakdown and amylose leaching occur before reaching the high viscosity levels that are seen in untreated native potato starch. Higher treatment temperatures further reduced peak viscosity (DHT 110 NPS vs. DHT 130 NPS). This shows that stronger disruption of crystalline order, evidenced by XRD and DSC results, further reduces the ability of treated starch granules to swell.

The pasting curves of dual-modified starches are shown in Figure 4 and Figure 5. When the predominant cation is monovalent, higher peak viscosities are observed, whereas the presence of divalent cations causes a suppression of peak viscosity. These observations can be explained by ionic cross-linking of starch chains via phosphate monoester groups and divalent cations. Interestingly, further differences between the monovalent ions as well as divalent ions were found. Figure 4 below shows that peak viscosities of dual-modified starches are decreasing in the order of DHT 110 NPS K > DHT 110 NPS Na > DHT 110 NPS > DHT 110 NPS Ca > DHT 110 NPS Mg. Similarly, DHT 130 NPS Ca and DHT 130 NPS Mg possess lower peak viscosities than DHT 130 NPS. Again, DHT 130 NPS Mg showed a lower peak viscosity than DHT 130 NPS Ca. Potentially, the more amorphous character of dry-heated starch allows these specific ion effects on granular swelling to be more visible due to increased starch-ion interactions and increased starch chain mobility.

Interestingly, the observed specific ion effects follow the Hofmeister series, which categorises ions based on their ability to stabilise or destabilise macromolecules (usually proteins) in solution: K^+^ < Na^+^ and Ca^2+^ < Mg^2+^ [33]. Following the reasoning of the Hofmeister series, the following hypothesis could be made: K^+^ has a larger ionic radius and is less strongly hydrated than Na^+^. Therefore, potassium ions might promote granular swelling and amylose leaching more strongly by reducing intermolecular hydrogen bonding in the amorphous region compared to sodium ions. Mg^2+^ has a smaller ionic radius with a more pronounced hydration shell than Ca^2+^, which may lead to stronger hydrogen bond stabilisation between starch chains in the amorphous regions, which restricts granular swelling and amylose leaching in addition to ionic cross-linking.

Previously, ion exchange in combination with annealing was reported by our group [18]. Notably, ion exchange at room temperature and ion exchange during annealing yielded no significant difference in pasting properties between samples containing predominantly Na^+^ and K^+^ or Mg^2+^ and Ca^2+^. In these annealing studies, the impact seems to be mainly based on the valency of the cations. Monovalent cations were included to contrast the ionic cross-linking effect of divalent cations. Since ion exchange and annealing left the starch structure unaltered or increased crystallinity, amorphisation might be important for specific ion effects to appear.

**Figure 6:**
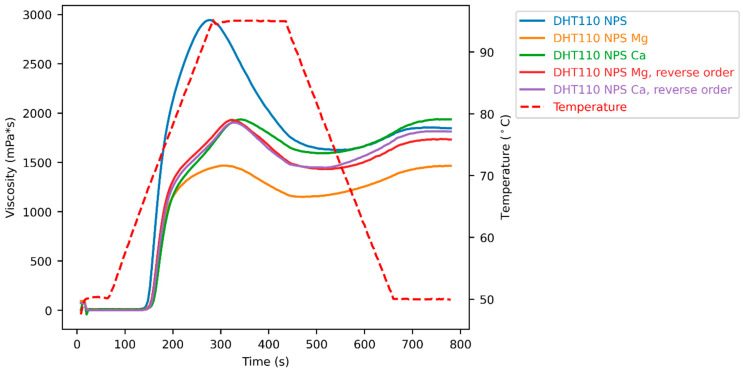
Pasting curves of experiments in reverse order.

To study the influence of the order of modifications, the dual modification was also carried out in reverse order, so that dry-heated native potato starch (DHT 110 NPS) was subjected to ion exchange with magnesium and calcium ions. ‘DHT 110 NPS Mg, reverse order’ and ‘DHT 110 NPS Ca, reverse order’ yield almost identical pasting curves (Figure 6, Table A1). However, they differ from DHT 110 NPS Mg. This means that the presence of Mg^2+^ ions during dry heat treatment in DHT 110 NPS Mg changes the swelling ability of the obtained starch granules to a different extent.

Thus, it is shown here that the order of dual modification is not interchangeable; this is in contrast to previous work showing that the impact on pasting properties was the same whether ion exchange was performed during annealing or whether ion exchange was performed as a second step after annealing [18].

**Figure A2:**
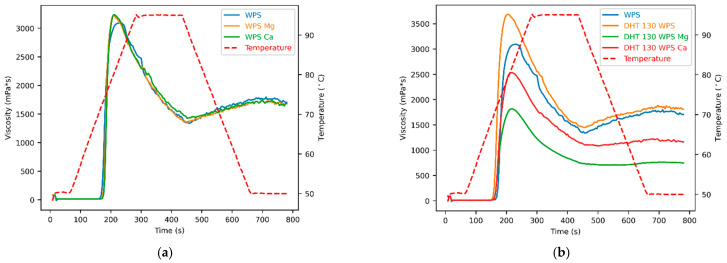
Pasting curves of waxy potato starch samples before (WPS) and after ion exchange with divalent cations (WPS Mg and WPS Ca) (**a**) and dry-heated waxy potato starch (DHT 130 WPS) and dual-modified waxy potato starch (DHT 130 WPS Mg and DHT 130 WPS Ca) (**b**).

Another piece of evidence hinting towards increased starch chain mobility through the presence of cations during DHT can be found in studies with waxy potato starch, which were conducted alongside the DHT 130 series (Figure A2). Waxy potato starch (WPS) was ion-exchanged with Mg^2+^ or Ca^2+^ ions and subsequently dry heat treated at 130 °C (equivalent to DHT 130 NPS Mg and DHT 130 NPS Ca). DHT 130 WPS shows a higher peak viscosity than untreated waxy potato starch. Again, lower peak viscosity was found in DHT 130 WPS Mg compared to DHT 130 WPS Ca (Figure A2). This is remarkable, since ion exchange at room temperature does not impact pasting properties of waxy potato starch due to the higher crystallinity and the absence of amylose (Figure A2).

The impact of DHT 110 and 130 on pasting properties matches with reports in the literature. Zhang et al. obtained native potato starches with overall lower viscosity after dry heat treatment at 130 °C for 3 h and 9 h, with the observed decrease being more severe after longer treatment times [31]. Liu et al. reported that dry heating of waxy potato starches at 110 °C for 0.5 to 2.5 h caused an increase in peak viscosity [23]. Slight amorphisation in waxy potato starch promotes starch swelling. This enables a stronger viscosity development, since untreated waxy starches have a more ordered structure due to the absence of amylose.

### 3.6. Digestibility

Nutritionally, starch is divided into rapid digestible starch (RDS), providing energy after 20 min of digestion, slowly digestible starch (SDS), which is digested after 2 h, and resistant starch (RS) that still resists enzymatic breakdown after 4 h and hence functions more like a dietary fibre [26,34]. Potato starch has a high resistant starch content due to its smooth granular surface without pores and surface channels, its B-type crystallinity, and the long chain length of amylose, among other characteristics [1,32]. Sevenou et al. suggested that potato starch shows a high degree of ordering in the granule periphery, causing a high resistance to enzyme hydrolysis [35]. Hydrothermal modifications induce changes in starch crystallinity and thereby alter susceptibility to enzymatic attack [11,31,36,37].

Table 6 summarises the impact of dry heat treatment with and without prior ion exchange. DHT caused an increase in the amount of digestible starch throughout the measurement period and a decrease in resistant starch content. This effect was more pronounced in DHT 130 NPS than in DHT 110 NPS. Thus, DHT leads to higher susceptibility to enzymatic digestion. DHT 130 NPS Mg and DHT 130 NPS Ca showed similar resistant and digestible starch contents as DHT 130 NPS. This suggests that the Mg- and Ca- enrichment prior to DHT 130 had no effect on starch digestibility.

The increase in starch digestibility after DHT 110 and DHT 130 is in line with the decrease in starch crystallinity seen in DSC and XRD measurements. Changes in digestibility through damaged granular surface (i.e., cracks, partial gelatinisation) were ruled out by SEM (Figure A1). The limited influence of ion exchange prior to DHT is in line with XRD data, which did not suggest an impact on starch crystallinity due to the presence of Mg or Ca during dry heat treatment. In this study, changes in crystalline structure were the main driver of changes in starch digestibility. Ions, which are mostly present in the amorphous regions, did not disturb starch crystalline order and therefore did not strongly alter starch digestibility.

According to studies by Noda et al., ion exchange of native potato starch with Mg or Ca does not change the digestibility of starch, which agrees with our findings [28]. Also consistent with our results, Liu et al. found a lower amount of resistant starch after dry heat treatment of waxy potato starch at 110 °C for 2.5 h. They attributed the observed changes to partial disruption of organised chain structures by DHT.

In a recent review, it was noted that the impact of DHT on starch digestibility varies with applied conditions, and thus, higher resistant starch contents have also been reported for dry-heated potato starch when starch crystallinity had increased [13]. Indeed, if relatively high moisture contents are employed during dry heat treatment, starches can be obtained that more closely resemble heat–moisture-treated starches.

## 4. Conclusions and Outlook

The present study adds to the understanding of starch–ion interactions by showcasing the role of specific ion effects in dry heat treatments of potato starches in addition to the previously described ionic cross-linking effect of divalent cations. The impact of cations on pasting properties was found to follow the Hofmeister series, with K^+^ > Na^+^ and Ca^2+^ > Mg^2+^ from higher to lower peak viscosities. With this, the versatility of dry heat treatment is increased, allowing greater flexibility in processing through the addition of a simple and low-cost ion-exchange step. Dry heat treatment and dual modification yielded starch products with lower crystallinity as evidenced by lower gelatinisation temperatures and broadening of X-ray diffractograms. The amount of total digestible starch increased through the induced changes in starch structure.

More easily digestible, functional carbohydrates combined with vital minerals may be of interest for the formulation of sports nutrition, such as in gels for endurance sports. Furthermore, these new insights may be applied to improve physical starch modifications with a targeted outcome. Indeed, an application study for improving a related commercial starch modification using this knowledge is currently underway. Finally, dual-modified starch that satisfies clean-label requirements and serves as an alternative for other chemically modified starches will find potential use in the food industry, as well in other market segments.

## Figures and Tables

**Table 1:** Standard measurement profile for RVA measurements.

Time (hh:mm:ss)	Function Type	Value
00:00:00	Temp	50 °C
00:00:00	Speed	960 rpm
00:00:10	Speed	160 rpm
00:01:00	Temp	50 °C
00:04:42	Temp	95 °C
00:07:12	Temp	95 °C
00:11:00	Temp	50 °C
00:13:00	End	

**Table 2:** Sample overview and dry heat treatment conditions. Dry heat treatment duration was 2 h in all cases.

Sample	Starting Material	Moisture Content of Starting Material	Treatment Temperature
DHT 110 NPS	NPS	7.1%	110 °C
DHT 110 NPS Mg	NPS Mg	6.4%	110 °C
DHT 110 NPS Ca	NPS Ca	6.4%	110 °C
DHT 110 NPS Na	NPS Na	6.1%	110 °C
DHT 110 NPS K	NPS K	6.2%	110 °C
DHT 130 NPS	NPS	2.8%	130 °C
DHT 130 NPS Mg	NPS Mg	2.0%	130 °C
DHT 130 NPS Ca	NPS Ca	2.4%	130 °C

**Table 3:** Mineral composition and amylose content of native potato starch and ion-exchanged starting materials. The mineral composition of the reverse experiments was measured after dual modification (i.e., dry heat treatment and subsequent ion exchange). Cation content of native potato starch (NPS) was measured in quintuple and phosphorus content in duplicate. Amylose contents were measured in duplicate.

Sample	Ca (ppm)	K (ppm)	Mg (ppm)	Na (ppm)	P (ppm)	Amylose (%)
NPS	167 ± 6	691 ± 3	45 ± 1	59 ± 4	748 ± 3	23.97 ± 0.02 *a*
NPS Mg	26	32	370	15	746	-
NPS Ca	665	9	8	13	727	-
NPS Na	106	17	22	607	741	-
NPS K	25	1130	6	17	722	-
DHT 130 NPS	164	709	76	75	764	23.84 ± 0.04 *a*
DHT 130 NPS Mg	77	34	391	32	727	24.36 ± 0.03 *a*
DHT 130 NPS Ca	679	42	22	35	745	24.12 ± 0.06 *a*
DHT 110 NPS Mg, reverse order	126	21	467	42	-	-
DHT 110 NPS Ca, reverse order	570	7	60	26	-	-

Values with the same letter in the same column are not significantly different (*p* > 0.05).

**Table 4:** Thermal properties of dry-heated starch samples. Samples were measured at least in duplicate.

Sample	Onset Temperature (°C)	Peak Temperature (°C)	Conclusion Temperature (°C)	Enthalpy of Gelatinisation (J/g)
NPS	60.39 ± 0.15 *a*	65.54 ± 0.18 *a*	70.92 ± 0.26 *a*	17.0 ± 0.2 *a*
DHT 110 NPS	56.16 ± 0.05 *e*	61.51 ± 0.06 *d*,*e*	69.00 ± 0.00 *b*,*c*	20.1 ± 1.1 *a*
DHT 110 NPS Mg	57.31 ± 0.00 *c*	62.52 ± 0.10 *b*,*c*	68.74 ± 0.17 *c*,*d*	20.45 ± 0.62 *a*
DHT 110 NPS Ca	57.85 ± 0.02 *b*	63.09 ± 0.01 *b*	69.48 ± 0.16 *b*	19.51 ± 0.04 *a*
DHT 110 NPS Na	56.67 ± 0.09 *d*	61.89 ± 0.12 *c*,*d*	68.13 ± 0.25 *d*,*e*	20.68 ± 3.82 *a*
DHT 110 NPS K	57.24 ± 0.09 *c*	62.16 ± 0.06 *c*,*d*	68.79 ± 0.02 *b*,*c*,*d*	18.66 ± 2.42 *a*
DHT 130 NPS	55.30 ± 0.18 *f*	60.90 ± 0.45 *e*	68.20 ± 0.25 *d*,*e*	16.4 ± 0.8 *a*
DHT 130 NPS Mg	56.04 ± 0.03 *e*	61.18 ± 0.11 *e*	67.03 ± 0.20 *f*	15.99 ± 0.55 *a*
DHT 130 NPS Ca	56.32 ± 0.12 *d*,*e*	61.07 ± 0.11 *e*	67.54 ± 0.19 *e*,*f*	18.09 ± 0.13 *a*

Values with the same letter in the same column are not significantly different (*p* > 0.05).

**Table 5:** Swelling power and solubility of dry-heated starches. Measurements were carried out in triplicate.

Sample	Swelling Power (g/g)		Solubility (%)	
	At 70 °C	At 90 °C	At 70 °C	At 90 °C
Native potato starch	11.71 ± 0.34 *d*	17.30 ± 0.60 *a*	1.08 ± 0.08 *c*	2.56 ± 0.32 *a*
DHT 110 NPS	13.28 ± 1.09 *b*,*c*	16.82 ± 0.13 *a*,*b*	1.54 ± 0.32 *c*	0.98 ± 0.44 *b*
DHT 110 NPS Mg	12.87 ± 0.76 *c*	15.10 ± 0.15 *b*,*c*	2.06 ± 0.54 *b*,*c*	1.15 ± 0.18 *b*
DHT 110 NPS Ca	12.88 ± 0.29 *c*	16.05 ± 0.51 *a*,*b*,*c*	1.35 ± 0.51 *c*	1.38 ± 0.40 *b*
DHT 110 NPS Na	15.24 ± 0.42 *a*	16.95 ± 1.72 *a*,*b*	1.65 ± 0.75 *c*	0.91 ± 0.15 *b*
DHT 110 NPS K	15.16 ± 0.43 *a*	16.36 ± 0.70 *a*,*b*	1.40 ± 0.20 *c*	1.28 ± 0.09 *b*
DHT 130 NPS	14.84 ± 0.15 *a*,*b*	17.93 ± 0.70 *a*	1.94 ± 0.11 *b*,*c*	1.35 ± 0.24 *b*
DHT 130 NPS Mg	13.26 ± 0.66 *b*,*c*	14.18 ± 0.38 *c*	3.69 ± 0.17 *a*	3.08 ± 0.16 *a*
DHT 130 NPS Ca	13.53 ± 0.18 *b*,*c*	17.68 ± 0.24 *a*	2.88 ± 0.25 *a*,*b*	2.54 ± 0.21 *a*

Values with the same letter in the same column are not significantly different (*p* > 0.05).

**Table 6:** Digestible and resistant starch contents of starch samples. Native potato starch (NPS) was measured in duplicate.

Sample	Digestible Starch at t0 [g/100 g Dry Sample]	Rapid Digestible Starch After 20 min [g/100 g Dry Sample]	Slowly Digestible Starch * After 2 h [g/100 g Dry Sample]	Total Digestible Starch After 240 min [g/100 g Dry Sample]	Resistant Starch After 240 min [g/100 g Dry Sample]
NPS	0.7 ± 0.1	2.5 ± 0.6	2.4 ± 0.1	6.7 ± 0.0	71.9 ± 1.3
DHT 110 NPS	2.3	2.6	8.1	12.2	65.0
DHT 130 NPS	3.1	6.5	11.5	18.1	62.1
DHT 130 NPS Mg	2.0	5.5	6.6	17.8	61.0
DHT 130 NPS Ca	1.9	6.6	5.6	15.8	65.2

* This value is subtracted by RDS.

## Data Availability

The original contributions presented in this study are included in the article. Further inquiries can be directed to the corresponding author.

## Appendix A

Changes in surface morphology were studied by scanning electron microscopy (SEM). Images were acquired following the procedure of Broekman et al. on a Fei NovaNanoSEM 650 using a CBS detector and an accelerating voltage of 5 kV. Samples were sputter-coated with a layer of gold [38].

**Table A1:** Pasting properties of starch samples. Native and waxy potato starches were measured in triplicate.

Samples	Peak Viscosity (mPa·s)	Trough Viscosity (mPa·s)	Breakdown (mPa·s)	Final Viscosity (mPa·s)	Setback (mPa·s)	Peak Time (min)	Pasting Temp (°C)
NPS	4036 ± 50.06	1596.9 ± 24.61	2439.1 ± 28.49	1864.3 ± 27.89	267.4 ± 16.86	3.6 ± 0.04	69.5 ± 0.05
DHT 110 NPS	2943	1625	1318	1846	221	4.6	66.90
DHT 110 NPS Mg	1469	1153	316	1465	312	5.13	68.50
DHT 110 NPS Ca	1935	1593	342	1937	344	5.67	69.45
DHT 110 NPS Na	3989	1780	2209	1965	185	4.07	67.05
DHT 110 NPS K	4343	1788	2555	1963	175	3.87	66.95
DHT 130 NPS	2091	1566	525	1833	267	4.93	66.95
DHT 130 NPS Mg	639	446	193	586	140	4.33	68.65
DHT 130 NPS Ca	1099	955	144	1417	462	5.40	68.50
DHT 110 NPS Mg, reverse order	1932	1432	500	1733	301	5.40	68.50
DHT 110 NPS Ca, reverse order	1903	1447	456	1813	366	5.47	68.60
Waxy potato starch	3116.5 ± 24.5	1372 ± 31	1744.5 ± 6.5	1717 ± 12	345 ± 19	3.87 ± 0	71.55 ± 0.4
WPS Mg	3222	1345	1877	1679	334	3.47	73.55
WPS Ca	3236	1423	1813	1681	258	3.53	73.55
DHT 130 WPS	3685	1450	2235	1810	360	3.40	69.45
DHT 130 WPS Mg	1819	704	1115	749	45	3.60	71.05
DHT 130 WPS Ca	2534	1090	1444	1166	76	3.60	70.95
